# Nonlytic exocytosis of *Cryptococcus neoformans* from neutrophils in the brain vasculature

**DOI:** 10.1186/s12964-019-0429-0

**Published:** 2019-09-09

**Authors:** Xiaofan Yang, Huijuan Wang, Fan Hu, Xichen Chen, Mingshun Zhang

**Affiliations:** 1 The Laboratory Center for Basic Medical Sciences, Nanjing Medical University, Nanjing, 211166 China; 2 Department of Immunology, Nanjing Medical University, Nanjing, 211166 China; 3 State Key Laboratory of Reproductive Medicine, Nanjing Medical University, Nanjing, 211166 China; 4 Analysis center, Nanjing Medical University, Nanjing, 211166 China; 5 NHC Key Laboratory of Antibody Technique, Nanjing Medical University, 101 Longmian Road, Nanjing, 211166 China

**Keywords:** *C. neoformans*, Neutrophils, Brain, Nonlytic exocytosis, *real-time* imaging

## Abstract

**Background:**

*Cryptococcus neoformans* (*C. neoformans*) is an encapsulated budding yeast that causes life-threatening meningoencephalitis in immunocompromised individuals, especially those with acquired immunodeficiency syndrome (AIDS). To cause meningoencephalitis, *C. neoformans* circulating in the bloodstream must first be arrested in the brain microvasculature. Neutrophils, the most abundant phagocytes in the bloodstream and the first leukocytes to be recruited to an infection site, can ingest *C. neoformans*. Little is known about how neutrophils interact with arrested fungal cells in the brain microvasculature.

**Methods:**

A blood-brain barrier (BBB) in vitro model was established. The interactions between neutrophils adhering to brain endothelial cells and fungi were observed under a live cell imaging microscope. A flow cytometry assay was developed to explore the mechanisms. Immunofluorescence staining of brain tissues was utilized to validate *the* in vitro phenomena.

**Results:**

Using real-time imaging, we observed that neutrophils adhered to a monolayer of mouse brain endothelial cells could expel ingested *C. neoformans* without lysis of the neutrophils or fungi in vitro, demonstrating nonlytic exocytosis of fungal cells from neutrophils. Furthermore, nonlytic exocytosis of *C. neoformans* from neutrophils was influenced by either the fungus (capsule and viability) or the neutrophil (phagosomal pH and actin polymerization). Moreover, nonlytic exocytosis of *C. neoformans* from neutrophils was recorded in brain tissue.

**Conclusion:**

These results highlight a novel function by which neutrophils extrude *C. neoformans* in the brain vasculature.

**Graphical abstract:**

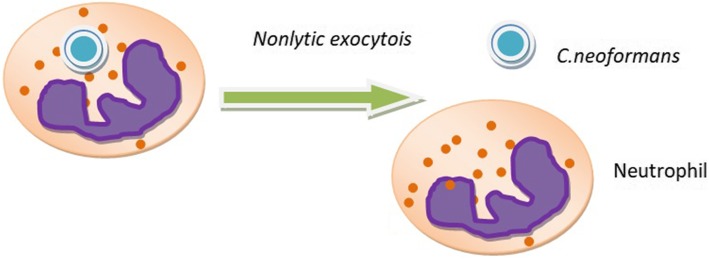

**Electronic supplementary material:**

The online version of this article (10.1186/s12964-019-0429-0) contains supplementary material, which is available to authorized users.

## Background

Cryptococcosis is an acquired immunodeficiency virus (AIDS)-defining opportunistic infection that also occurs in organ transplant recipients and cancer patients [1]. Globally, it is estimated that there are an estimated 223,100 cases of cryptococcosis and approximately 181,100 deaths from human immunodeficiency virus (HIV)-associated cryptococcal disease [2]. Additionally, cryptococcosis is also an infrequent fungal infection in patients with systemic lupus erythematosus [3] or tuberculosis [4, 5]. The causative microorganism is the encapsulated fungus *Cryptococcus neoformans*. Fungal cells are believed to enter the body through the respiratory tract and initially cause pneumonia. The most devastating event occurs when fungi disseminate into the brain via the bloodstream, consequently resulting in fatal meningoencephalitis.

Fungemia is often detected in AIDS patients during cryptococcosis [6]. Although extrapulmonary dissemination appears to be macrophage-associated [7], free fungal cells have been detected in the bloodstream [8]. These circulating fungal cells could be directly derived from the lung as free fungus or released from macrophages, as recent evidence has shown that macrophages can expel ingested *C. neoformans* via nonlytic exocytosis [9–11].

Recently, brain invasion by *C. neoformans* has been visualized in a mouse model based on intravital microscopy, and an important series of events that occur prior to transmigration into the brain has been postulated [12, 13]. The critical steps include fungal arrest in the vasculature of the brain and interaction and signaling of the fungal and endothelial cells leading to transmigration [12, 13]. As a result of these processes, the arrested fungal cells remain within the brain vasculature for hours, providing the opportunity for immune cells circulating in the bloodstream to recognize the fungal cells. However, the intravascular interactions of immune cells with the arrested fungal cells are largely unknown. This knowledge gap limits our advances in the prevention and treatment of the illness.

Neutrophils, one of the major players during infection, are typically the first immune cells to be recruited to an infection site and are capable of eliminating microorganisms by multiple means [14]. There is evidence that neutrophils play roles in protecting the host against *C. neoformans* [15]*.* In vitro, neutrophils internalize *C. neoformans* following opsonization with complement and antibody [16–18]. In vivo, neutrophils ingest *C. neoformans* in the lungs of mice following intratracheal infection [19] and kill the fungi with the aid of complement C5a in the brain vasculature [20]. Previously, we reported that neutrophils may ingest and remove *C. neoformans* from brain vessels [21]. In this study, we recorded neutrophils expelling *C. neoformans* without causing lysis of either the neutrophils or fungi, which may be regulated by fungal virulence and neutrophil actin polymerization.

## Materials and methods

### Animals

C57BL/6 J mice from the Laboratory Animal Center, Yangzhou University (Yangzhou, China) were housed under a 12 h light/dark cycle in specific pathogen-free conditions with free access to mouse chow and water. Animal experiments were approved by the Laboratory Animal Ethics Committee of Nanjing Medical University (1708004).

### *C. neoformans*

*C. neoformans* strain H99 was obtained from ATCC (catalog number 208821). *C. neoformans* B3501, Cap67, and Cap59 strains were gifts from Dr. Min Chen from the Second Military Medical University. The organisms were cultured in Sabouraud’s dextrose broth (Difco) at 32 °C with gentle rotation for 16 h and then washed 3 times in sterile PBS (pH 7.4) before use.

### Nonlytic exocytosis in vitro observed under a confocal microscope

Neutrophils were purified from bone marrow cells using a Percoll density gradient [22]. To determine the purity of neutrophils isolated from the bone marrow, the isolated cells were stained with PE anti-mouse F4/80 mAb (clone BM8, eBioscience) and Alexa Fluor 647 anti-mouse Gr-1 mAb (clone RB6-8C5, Invitrogen). As a control, cells were incubated with the corresponding isotype control antibodies. Antibodies and their ligands are listed in Additional file 1: Table S1.

To observe the dynamic interactions of neutrophils with the ingested *C. neoformans*, neutrophils purified from bone marrow cells were incubated with Alexa Fluor 647 anti-Gr-1 mAb (RB6-8C5, Invitrogen) and PE anti-mouse F4/80 mAb (BM8, eBioscience) for 30 min. The prestained neutrophils (5 × 10^6^) and *C. neoformans* labeled with FITC (5 × 10^7^) were added to the surface of a monolayer of mouse brain endothelial cells bEnd.3 (CRL-2299, ATCC) in a glass-bottom dish (P35G-1.5-10, MatTek), which represented an in vitro BBB model [23]. E1 mAb specific for cryptococcal polysaccharide (a gift from Dr. F. Dromer, Paris, France) was added to the culture, and the cells were incubated for 1 h in the live cell culture equipment of the confocal microscope (LSM510, Zeiss). The dish was then extensively washed 3 times with complete medium to remove most of the nonadherent neutrophils and *C. neoformans*. The interactions of neutrophils with the ingested *C. neoformans* were imaged, and time-lapse videos were taken using a confocal microscope.

### Flow cytometry analysis of nonlytic exocytosis

Flow cytometry analysis of nonlytic exocytosis was first developed by André Moraes Nicola and colleagues [11]. Briefly, neutrophils were purified from bone marrow [22] with a neutrophil isolation kit (130–097-658, Millipore) and stained with DDAO-SE (0.5 μM, Invitrogen) in PBS for 10 min and washed 3 times. *C. neoformans* were stained with FITC (1 mg/ml, Sigma-Aldrich) in PBS for 15 min and washed 3 times. Furthermore, neutrophils were stained with 7-AAD (1 μg/ml, Invitrogen) for 5 min to detect cell death, and *C. neoformans* were stained with Uvitex 2B (0.01%, Polysciences Inc.) in PBS for 1 min to detect fungi outside of neutrophils (Additional file 2: Figure S1). DDAO-SE-stained neutrophils and FITC-stained H99 (neutrophils:H99 = 1:1) cells were incubated in the presence of the *C. neoformans*-specific anti-E1 antibody (1 μg/ml) for 2 h and further stained with Uvitex-2B and 7-AAD. Subsequently, FITC^+^DDAO-SE^+^ 7-AAD^−^Unvitex2B^−^ cells, which represented live neutrophils containing *C. neoformans*, were flow sorted with a BD FACS ARIA II SORP. Immediately after sorting, the cells were analyzed for purity, and 1 × 10^6^ sorted neutrophils in 1 ml of complete medium (10% fetal calf serum in RPMI 1640 medium) were incubated in the well of a 24-well plate with or without chemicals for 2 h. After incubation, the cells were harvested and stained with 7-AAD again for flow cytometry analysis. FITC^−^DDAO-SE^+^ 7-AAD^−^ cells were considered live neutrophils that had expelled the ingested *C. neoformans*. As described by Moraes Nicola and colleagues [11], the rate of nonlytic exocytosis was calculated based on the postsorted purity and postcultured live neutrophils not associated with *C. neoformans*.

### Immunofluorescence staining of brain tissues

Mice were euthanized and perfused 3 h after *i.v*. infection by tail vein with 20 × 10^6^ H99 cells, and the brain was removed for immunofluorescence staining. To avoid contamination of tissues by circulating fungi, perfusion was performed by injecting sterile saline (50 ml) into the left ventricle, and the right atrium was cut open to allow drainage during the procedure [24]. The brain tissues of infected mice were prepared for frozen sections, as described previously [13]. In brief, the tissues were removed and frozen in OCT compound. Frozen tissue blocks were cut on a cryostat microtome, and 5-μm sections were placed on coated glass slides. Tissue sections were fixed in 2% neutral buffered paraformaldehyde (PFA) for 10 min. Sections were then incubated with 2% goat serum in PBS, followed by incubation with rabbit-anti-mouse collagen IV (PA1–26148, Invitrogen), mouse-anti-cryptococcal polysaccharide (E1, a gift from Dr. F. Dromer, Paris, France), and rat-anti-mouse Ly6G (clone 1A8, Biolegend) at 4 °C overnight. After 3 washes, sections were incubated for 30 min with Alex Fluor 647 goat-anti-rabbit IgG (H + L) (A-21244, Invitrogen) to delineate brain microvasculature, Alexa Fluor 488 goat-anti-mouse IgG (H + L) (A-11001, Invitrogen) to identify *C. neoformans*, and Alexa Flour 555 goat-anti-rat IgG (H + L) (A-21434, Invitrogen) to identify neutrophils in the brain. The sections were rinsed and mounted with glycerol and checked under a confocal microscope.

### Statistical analysis

Data are expressed as the mean ± SEM. An ANOVA was performed to establish equal variance, and a 2-tailed Student’s t test with Bonferroni correction was applied to determine statistical significance, which was defined as *p* < 0.05.

## Results

### Nonlytic exocytosis of *C. neoformans* from neutrophils in vitro

**Fig. 1:**
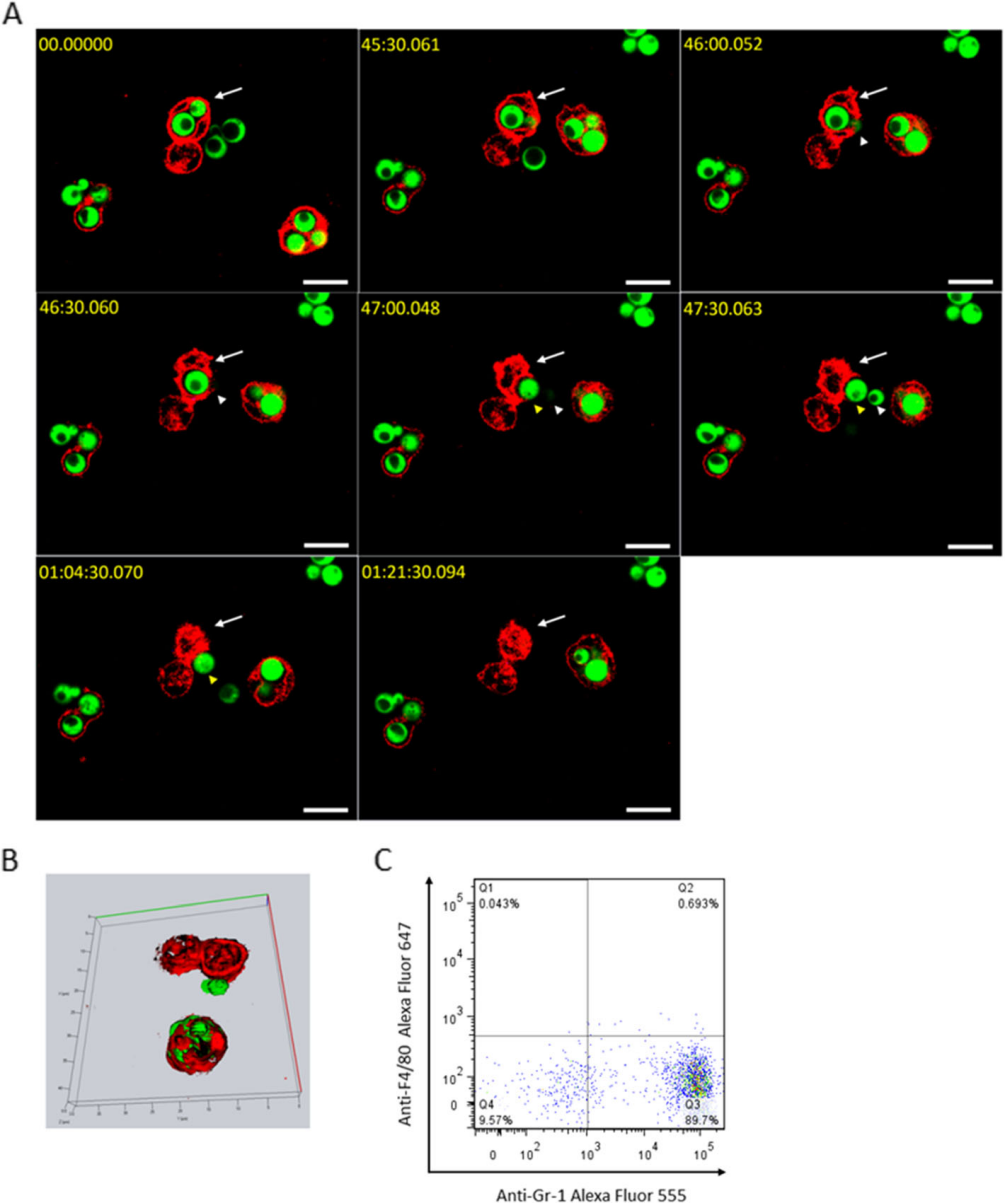
Nonlytic exocytosis of *C. neoformans* by neutrophils demonstrated by time-series images and a 3D reconstruction of z-stack images. **a** Time-series images. *C. neoformans* H99 cells labeled with FITC were incubated with neutrophils, which were prestained with anti-Gr-1 (pseudocolored red) and anti-F/80 (pseudocolored blue), in a glass-bottomed dish with a precoated monolayer of mouse brain endothelial bEnd.3 cells in the presence of anti-capsule antibody E1. After 1 h of incubation, nonadherent cells were removed. Multichannel time-lapse fluorescence images were taken to record the dynamic process of nonlytic exocytosis of *C. neoformans* by the neutrophil (marked by an arrow), capturing one frame every 30 s for 3 h or until the occurrence of expulsion. At the start of image acquisition, the neutrophil contained two fungal cells. Forty-six minutes later, the phagosome fused with the cell membrane. The two fungal cells (marked by arrowheads) escaped from the neutrophil within 1 min. **b** A 3D image reconstruction. To verify the expulsion of *C. neoformans*, z-stack images were taken at the end of the image acquisition period (81 min after the microscopy observation began) for the reconstruction of a 3D image of neutrophils and fungi. The neutrophils did not contain fungi; the two expelled fungal cells were located beside the “empty” neutrophil. Bar, 5 μm. **c** Flow cytometry analysis of neutrophils. Neutrophils purified from bone marrow were stained with Alexa Flour 647 anti-F4/80 and Alexa Flour 555 anti-Gr-1 or isotype control antibodies. Based on the flow cytometry analysis, approximately 90% of cells were F4/80^−^Gr-1^+^ (neutrophils), and less than 1% of cells were F4/80^+^Gr-1^−^ (resident monocytes) or F4/80^+^Gr-1^+^ (inflammatory monocytes), indicating that the vast majority of Gr-1-positive cells in the cell preparation were neutrophils. Representative results from 3 repeated experiments

To address how neutrophils interact with the ingested *C. neoformans,* we first established an in vitro BBB model using the mouse brain microvasculature endothelial cell line bEnd.3. Neutrophils containing *C. neoformans* were seeded and attached to a monolayer of endothelial cells. Initially, we observed neutrophils containing fungi in the phagosome (Fig. 1a). At 46 min of image acquisition, one neutrophil started to expel the ingested fungal cell. The small yeast cell was first expelled, and the large yeast cell was also expelled in 1 min. To confirm the exocytosis of *C. neoformans* by neutrophils without lysis, we extended our observation (Fig. 1a, Additional file 4: Movie S1) and reconstructed a z-stack of images at the end of the acquisition period. As shown in the 3D reconstruction image (Fig. 1b) and movie (Additional file 5: Movie S2), the neutrophil that contained the two fungal cells did not harbor any fungi; the two fungal cells were located close to but outside of the neutrophil. More importantly, both the neutrophils and fungi were intact. Taken together, our results demonstrate that neutrophils expel intracellular *C. neoformans* without lysis of both the cell itself and the fungus. Interestingly, two neighboring neutrophils containing *C. neoformans* shown in Fig. 1a, b and Additional file 4: Movie S1 did not undergo nonlytic exocytosis. Indeed, in our repeated observations (n > 20), nonlytic exocytosis was recorded in approximately 5% of neutrophils containing *C. neoformans*. Notably, less than 1% of purified cells were F4/80 positive based on the flow cytometry analysis (Fig. 1c).

### Fungal capsule and viability influence nonlytic exocytosis

**Fig. 2:**
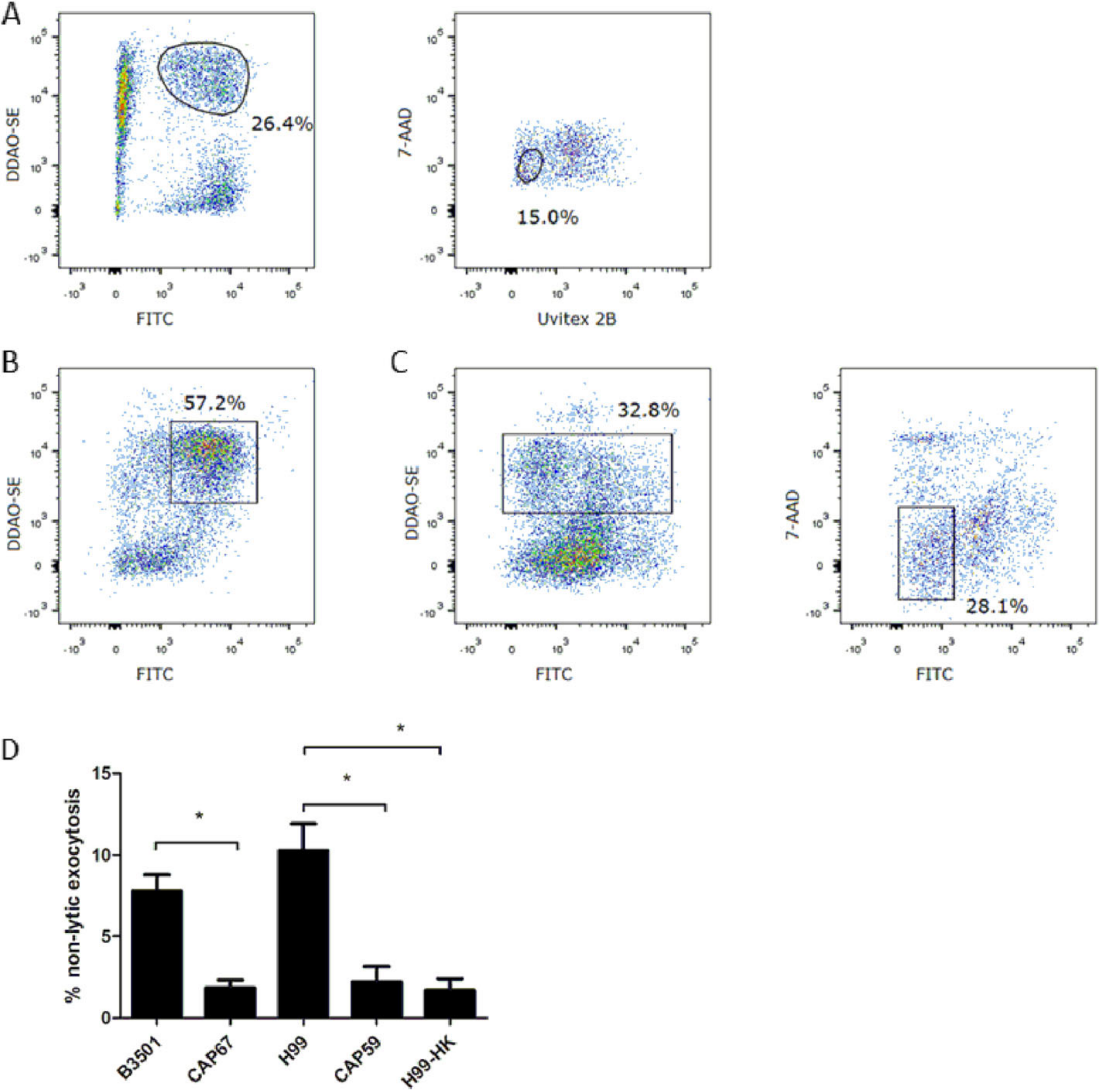
Flow cytometry analysis of nonlytic exocytosis. **a** After incubation, live neutrophils with intracellular *C. neoformans* (FITC^+^DDAO-SE^+^ 7-AAD^−^Unvitex2B^−^) were sorted. **b** Immediately after sorting, the cells were analyzed for purity. **c** After 2 h of incubation with or without drugs, neutrophils with *C. neoformans* were collected, stained with 7-AAD and further analyzed. FITC^−^DDAO-SE^+^ 7-AAD^−^ cells were considered neutrophils undergoing nonlytic exocytosis. Representative results of 5 repeated experiments. **d** Fungal capsule and viability regulate nonlytic exocytosis. Sorted live neutrophils containing either wild-type *C. neoformans* (B3501, H99) or acapsular strains (Cap67, Cap59) were incubated for 2 h. Nonlytic exocytosis was calculated as described by André Moraes Nicola, mBio, 2011. Heat-killed H99 cells were included to explore whether viability affects nonlytic exocytosis. *, *p* < 0.05

Microscopy observations are time-consuming and ineffective in the quantification analysis. André Moraes Nicola and colleagues developed a flow cytometric method to explore the mechanisms of nonlytic exocytosis of *C. neoformans* from macrophages [11]. Accordingly, we sorted live neutrophils containing *C. neoformans* (FITC^+^DDAO-SE^+^ 7-AAD^−^Unvitex2B^−^, Fig. 2a) and calculated the frequency of nonlytic exocytosis, in which neutrophils expelled phagocytosed *C. neoformans* and became FITC negative (Fig. 2b–c). The capsule plays a vital role in the pathogenesis of *C. neoformans* infection, as acapsular strains are usually hypovirulent. In neutrophils incubated with the acapsular strain Cap67, the occurrence of nonlytic exocytosis was significantly decreased compared with that in neutrophils incubated with the wild-type B3501 strain. Similarly, nonlytic exocytosis of the acapsular strain Cap59, was also significantly reduced compared with that of the wild-type strain H99, suggesting that the capsule may regulate the nonlytic exocytosis of *C. neoformans* from neutrophils. Neutrophils killed acapsular strains more effectively than capsulated strains. Therefore, we hypothesized that fungal viability may affect nonlytic exocytosis from neutrophils. As previously reported, nonlytic exocytosis of heat-killed *C. neoformans* from macrophages is significantly suppressed [9, 10], and we observed that the expulsion of heat-killed *C. neoformans* from neutrophils was markedly decreased (Fig. 2d). In summary, nonlytic exocytosis of *C. neoformans* from neutrophils is regulated by the fungal capsule and viability.

### Roles of phagosome and actin in nonlytic exocytosis

**Fig. 3:**
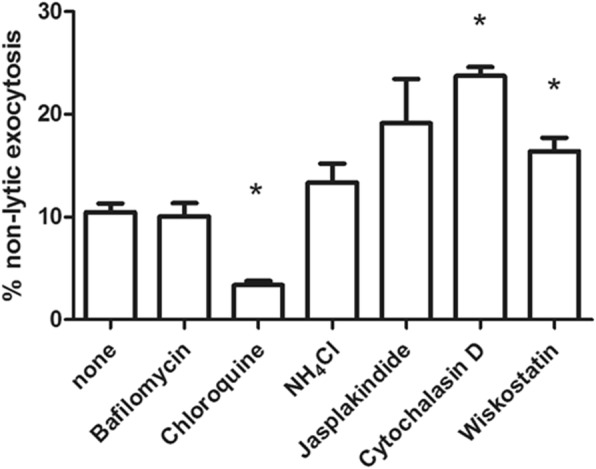
Phagosomes and actin polymerization regulate nonlytic exocytosis. Live neutrophils containing H99 cells were treated with various chemicals for 2 h, and the rate of nonlytic exocytosis was calculated. Bafilomycin A1 (100 nM), vacuolar ATPase inhibitor; chloroquine (10 μM) and ammonium chloride (20 mM), weak bases; jasplakinolide (50 nM), actin depolymerization inhibitor; cytochalasin (10 nM), actin polymerization inhibitor; and wiskostatin (100 nM), Arp2/3 complex-mediated actin polymerization inhibitor. *, *p* < 0.05

After phagocytosis, phagosomes containing the pathogens usually fuse with lysosomes and form lysophagosomes, in which pathogens are destroyed by various enzymes. As observed in Fig. 1, phagosomes containing fungi fused with the cell membrane before nonlytic exocytosis. The vacuolar ATPase inhibitor bafilomycin A1, which pumps protons into the phagosome and accelerates its acidification, barely changed the frequency of nonlytic exocytosis. Treatment with the weak base chloroquine, which helps phagosome neutralization, significantly decreased the rate of nonlytic exocytosis. Since ammonium chloride, another weak base, plays an insignificant role in nonlytic exocytosis, we hypothesized that chloroquine may regulate nonlytic exocytosis via a phagosome-independent pathway. For example, chloroquine may exert fungicidal effects [25, 26], as evidenced by the significant increase in cytotoxicity of neutrophils against *C. neoformans* with the aid of chloroquine (Additional file 3: Figure S2). Thereafter, the dead *C. neoformans* killed by chloroquine might not be expelled from neutrophils (Fig. 3).

As observed in Fig. 1, neutrophils expelled *C. neoformans* cells in 2 min. We therefore hypothesized that neutrophil actin polymerization may contribute to the nonlytic exocytosis of *C. neoformans*. In the expulsion of *C. neoformans* from macrophages, the actin flash was inversely correlated with nonlytic exocytosis [27]. Therefore, we tested whether chemicals interfering with actin flashes play roles in nonlytic neutrophil exocytosis. As shown in Fig. 3, treatment with the actin polymerization inhibitor cytochalasin D significantly enhanced the nonlytic exocytosis of *C. neoformans* from neutrophils. Moreover, treatment with the Arp2/3 complex-mediated actin polymerization inhibitor wiskostatin also increased *C. neoformans* expulsion from neutrophils, although to a lesser extent. In contrast, treatment with jasplakinolide, a potent actin polymerization inducer, had a reduced effect on the nonlytic exocytosis. Collectively, these results show that classical WASP-Arp2/3 complex-mediated actin filament nucleation may play a role in the nonlytic exocytosis of *C. neoformans* from neutrophils.

### Nonlytic exocytosis of *C. neoformans* from neutrophils in the brain vasculature

**Fig. 4:**
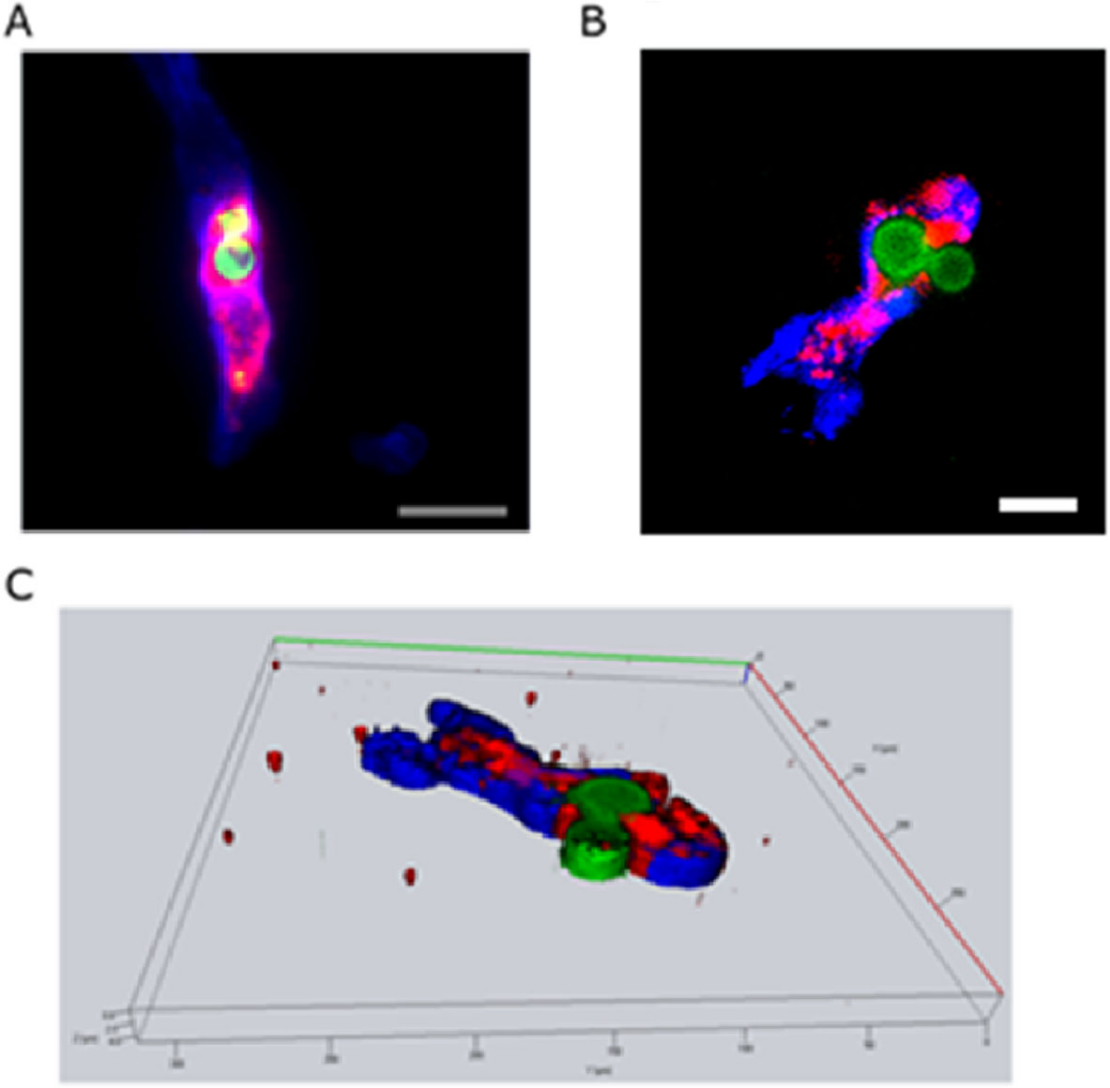
Neutrophils expel *C. neoformans* in the brain vasculature. Neutrophils were stained with anti-Ly6G (pseudocolored red); *C. neoformans* was stained with the anti-capsule antibody E1 (pseudocolored green), and the brain vasculature was stained with anti-collagen IV (pseudocolored blue). **a** Neutrophils containing *C. neoformans* in the brain vasculature; original scale bar, 20 μm; **b** Two fungal cells located in a neutrophil attached to a brain capillary. One fungal cell seemed to be expelled from the neutrophil; original scale bar, 10 μm. **c** A 3D reconstructed image based on z-stack images was captured for further analysis

To determine whether neutrophils recognize *C. neoformans* in the brain in vivo, we first recorded the intravascular interactions of neutrophils with *C. neoformans* in the brains of living mice using intravital microscopy. Gr-1 is predominantly expressed on mouse neutrophils and is also expressed on inflammatory monocytes [28]. However, the vast majority of Gr-1^+^ cells in the circulation are neutrophils, which are the most abundant white blood cell type in circulation. Therefore, anti-Gr-1 mAbs have been extensively used to label mouse neutrophils in intravital imaging [29]. Nevertheless, in this study, we also injected the mice with anti-F4/80 mAb to exclude the tiny population of Gr-1^+^ monocytes from neutrophils, as Gr-1^+^ monocytes also express F4/80. To determine whether neutrophils expel *C. neoformans* in the brain in vivo, we recorded the intravascular interactions of neutrophils with *C. neoformans* in the brains using immunohistochemistry. As shown in Fig. 4a, intravascular neutrophils engulfed *C. neoformans* in the brain. As expected, a neutrophil attached to the brain capillary expelled *C. neoformans* (Fig. 4b-c, Additional file 6: Movie S3). Therefore, we provide direct evidence that nonlytic exocytosis of *C. neoformans* from neutrophils occurs in the brain vasculature.

## Discussion

As the most commonly encountered clinical manifestation of cryptococcosis, cryptococcal meningoencephalitis is a devastating infectious disease. In AIDS patients, the mortality rate for cryptococcal meningoencephalitis was approximately 20% [30]. Once arrested in the brain vasculature, *C. neoformans* may transmigrate across the BBB and proliferate in the brain parenchyma. Therefore, it is imperative to explore how immune cells respond to *C. neoformans* in the brain [31]. Depletion of phagocytes, including macrophages and neutrophils, may impair the infiltration of *C. neoformans* into the brain [32]. It has been established that macrophages [9–11] and monocytes [33] can expel intracellular *C. neoformans*. In the present study, we documented that nonlytic exocytosis of *C. neoformans* from neutrophils occurs in vitro and in vivo.

Neutrophils and macrophages originate from common myeloid progenitors. Neutrophils, however, are different from macrophages. First, neutrophils are considered key responders to infection. Once released into the bloodstream, *C. neoformans* transmigrate across the BBB within 6 h [24]. Therefore, an immediate immune response is indispensable for the clearance or dissemination of *C. neoformans* brain infections. Indeed, intravascular clearance of *C. neoformans* is largely dependent on neutrophils [20]. Second, the half-life of neutrophils is much shorter than that of macrophages. In ex vivo culture, neutrophils may die within 24 h. The average lifespan for circulating neutrophils, however, is as long as 5.4 days in vivo [34], raising the possibility that neutrophils may not be as fragile as expected. In the present study, neutrophils cocultured with endothelial cells and *C. neoformans* were observed and analyzed within several hours. As microbes may regulate the aging of neutrophils [35], we hypothesize that *C. neoformans* may affect the lifespan of neutrophils. Third, the neutrophil phagosome may be acidification independent [36]. In the nonlytic exocytosis of *C. neoformans* from macrophages [37], phagosomal pH modulation was found to be associated with exocytosis. In neutrophils, phagosomal pH modification, either with vacuolar ATPase inhibitor bafilomycin A1 or ammonium chloride treatment, had little effect on the nonlytic exocytosis of *C. neoformans.* The effect of chloroquine on the occurrence of nonlytic exocytosis was largely related to the regulation of fungal viability rather than phagosomal acidification. Similar observations were observed for the capsule-deficient strains or heat-killed fungi.

Previously, we demonstrated that neutrophils may phagocytose arrested *C. neoformans* attached to the brain vasculature, which may help alleviate brain infections [21]. In this study, we provide evidence that neutrophils expel intracellular *C. neoformans* in vitro and in the brain vasculature. We hypothesize that the role of neutrophils may be a double-edged sword, i.e., both protective and deleterious, during the course of cryptococcosis. On the one hand, neutrophils are important for killing [16] or removing *C. neoformans* arrested in the brain microvasculature; on the other hand, neutrophils carry *C. neoformans* and exocytose them in the brain vasculature, contributing to the brain infection.

Our study has some notable limitations. The nonlytic exocytosis of *C. neoformans* from monocytes and macrophages may originate from environmental interactions [38, 39]. In contrast, the evolutionary role of nonlytic exocytosis of *C. neoformans* from neutrophil is unknown. Moreover, neutrophils are heterogeneous with many different functions [40]. Neutrophils with nonlytic exocytosis capabilities and the mechanisms warrant improved definitions.

## Conclusion

Our results suggest that neutrophils adhering to brain vasculature extrude *C. neoformans* via nonlytic exocytosis, which is regulated by fungal virulence factors (viability and capsule) and neutrophils (phagosome and actin). The mechanisms behind the nonlytic exocytosis of *C. neoformans* from neutrophils in the brain vasculature require further study.

## Additional files

Additional file 1: **Table S1.** Antibodies in the study. (DOCX 14 kb)

Additional file 2: **Figure S1.** Flow cytometry analysis of neutrophils and *C. neoformans* stained with various fluorophores. Bone marrow-derived neutrophils were stained with DDAO-SE (0.5 μM, Invitrogen) in PBS for 10 min and washed 3 times. *C. neoformans* cells were stained with FITC (1 mg/ml, Sigma-Aldrich) in PBS for 15 min and washed 3 times. Furthermore, neutrophils were stained with 7-AAD (1 μg/ml, Invitrogen) for 5 min to detect cell death, and *C. neoformans* were stained with Uvitex 2B (0.01%, Polysciences Inc.) in PBS for 1 min to detect extracellular fungi. Representative results of 5 repeated experiments. (TIF 575 kb)

Additional file 3: **Figure S2.** Chloroquine increased the fungicidal activity of neutrophils against *C. neoformans*. In a 96-well plate, *C. neoformans* (5 × 10^3^ cells per well) were incubated alone or with neutrophils (1 × 10^5^ cells per well) and chloroquine (10 μM, working concentration) for 4 h. After incubation, the live fungi were quantified on YPA agar. *, *p* < 0.05. (TIF 235 kb)

Additional file 4: **Movie S1.** FITC-labeled *C. neoformans* H99 were incubated with anti-Gr-1 (pseudocolored red) and anti-F4/80 (pseudocolored blue) prestained neutrophils in a glass-bottomed dish with a precoated monolayer of mouse brain endothelial bEnd.3 cells in the presence of anti-capsule antibody E1. After 1 h of incubation, nonadherent cells were removed. Multichannel time-lapse fluorescence images (oil-immersion objective lens) were captured to record the dynamic process of nonlytic exocytosis of *C. neoformans* by the neutrophil (marked by an arrow), with one frame captured every 30 s for 3 h or until the occurrence of expulsion. The time-series images were exported as a video at 5 frames per second. Also refer to Fig. 1. (MOV 1612 kb)

Additional file 5: **Movie S2.** A 3D reconstruction from z-stack images taken at the end of the image acquisition period (81 min after the microscopy observation began) showing the “empty” neutrophil (pseudocolored red) and the two expelled fungal cells (pseudocolored green) beside the neutrophil. Also refer to Fig. 1. (MOV 8570 kb)

Additional file 6: **Movie S3.** A 3D movie reconstructed from z-stack images of immunofluorescence stained cells showing the nonlytic exocytosis of *C. neoformans* from neutrophils in the brain vasculature. Also refer to Fig. 4. (MOV 2676 kb)

## Data Availability

All data generated or analyzed during this study are included in this published article (and its Supplementary Information files).

## Abbreviations

AIDS: Acquired immunodeficiency syndrome
BBB: Blood-brain barrier
*C. neoformans*: *Cryptococcus neoformans*
HIV: Human immunodeficiency virus
PFA: Paraformaldehyde

## References

[1] Kwon-Chung KJ, Sorrell TC, Dromer F, Fung E, Levitz SM (2000). Cryptococcosis: clinical and biological aspects. Med Mycol.

[2] Rajasingham R, Smith RM, Park BJ, Jarvis JN, Govender NP, Chiller TM (2017). Global burden of disease of HIV-associated cryptococcal meningitis: an updated analysis. Lancet Infect Dis.

[3] Fang W, Chen M, Liu J, Hagen F, Ms A, Al H (2016). Cryptococcal meningitis in systemic lupus erythematosus patients: pooled analysis and systematic review. Emerg Microbes Infect.

[4] Mete B, Saltoglu N, Vanli E, Ozkara C, Arslan F, Mert A (2016). Simultaneous cryptococcal and tuberculous meningitis in a patient with systemic lupus erythematosus. J Microbiol Immunol Infect.

[5] Chen M, Al-Hatmi AM, Chen Y, Ying Y, Fang W, Xu J (2016). Cryptococcosis and tuberculosis co-infection in mainland China. Emerg Microbes Infect..

[6] Chretien F, Lortholary O, Kansau I, Neuville S, Gray F, Dromer F (2002). Pathogenesis of cerebral Cryptococcus neoformans infection after fungemia. J Infect Dis.

[7] Casadevall A (2010). Cryptococci at the brain gate: break and enter or use a Trojan horse?. J Clin Investig.

[8] Santangelo R, Zoellner H, Sorrell T, Wilson C, Donald C, Djordjevic J (2004). Role of extracellular phospholipases and mononuclear phagocytes in dissemination of cryptococcosis in a murine model. Infect Immun.

[9] Alvarez M, Casadevall A (2006). Phagosome extrusion and host-cell survival after Cryptococcus neoformans phagocytosis by macrophages. Curr Biol.

[10] Ma H, Croudace JE, Lammas DA, May RC (2006). Expulsion of live pathogenic yeast by macrophages. Curr Biol.

[11] Nicola AM, Robertson EJ, Albuquerque P, Derengowski Lda S, Casadevall A. Nonlytic exocytosis of Cryptococcus neoformans from macrophages occurs in vivo and is influenced by phagosomal pH. MBio. 2011;2(4):e00167–11. https://www.ncbi.nlm.nih.gov/pmc/articles/PMC3150755/. 10.1128/mBio.00167-11 PMC3150755 21828219

[12] Shi M, Colarusso P, Mody CH (2012). Real-time in vivo imaging of fungal migration to the central nervous system. Cell Microbiol.

[13] Shi M, Li SS, Zheng C, Jones GJ, Kim KS, Zhou H (2010). Real-time imaging of trapping and urease-dependent transmigration of Cryptococcus neoformans in mouse brain. J Clin Invest.

[14] Hickey MJ, Kubes P (2009). Intravascular immunity: the host-pathogen encounter in blood vessels. Nat Rev Immunol.

[15] Zhang M, Sun D, Shi M (2015). Dancing cheek to cheek: Cryptococcus neoformans and phagocytes. Springerplus..

[16] Kozel TR, Highison B, Stratton CJ (1984). Localization on encapsulated Cryptococcus neoformans of serum components opsonic for phagocytosis by macrophages and neutrophils. Infect Immun.

[17] Voelz K, May RC (2010). Cryptococcal interactions with the host immune system. Eukaryot Cell.

[18] Sun D, Zhang M, Liu G, Wu H, Zhu X, Zhou H (2015). Real-time imaging of interactions of neutrophils with Cryptococcus neoformans demonstrates a crucial role of complement C5a-C5aR signaling. Infect Immun.

[19] Feldmesser M, Kress Y, Novikoff P, Casadevall A (2000). Cryptococcus neoformans is a facultative intracellular pathogen in murine pulmonary infection. Infect Immun.

[20] Sun D, Zhang M, Liu G, Wu H, Li C, Zhou H (2016). Intravascular clearance of disseminating Cryptococcus neoformans in the brain can be improved by enhancing neutrophil recruitment in mice. Eur J Immunol.

[21] Zhang M, Sun D, Liu G, Wu H, Zhou H, Shi M (2016). Real-time in vivo imaging reveals the ability of neutrophils to remove Cryptococcus neoformans directly from the brain vasculature. J Leukoc Biol.

[22] Boxio R, Bossenmeyer-Pourie C, Steinckwich N, Dournon C, Nusse O (2004). Mouse bone marrow contains large numbers of functionally competent neutrophils. J Leukoc Biol.

[23] Koto T, Takubo K, Ishida S, Shinoda H, Inoue M, Tsubota K (2007). Hypoxia disrupts the barrier function of neural blood vessels through changes in the expression of claudin-5 in endothelial cells. Am J Pathol.

[24] Charlier C, Chretien F, Baudrimont M, Mordelet E, Lortholary O, Dromer F (2005). Capsule structure changes associated with Cryptococcus neoformans crossing of the blood-brain barrier. Am J Pathol.

[25] Harrison TSGG, Levitz SM (2000). Conditional lethality of the diprotic weak bases chloroquine and quinacrine against *Cryptococcus neoformans*. J Infect Dis.

[26] Levitz SM, Harrison TS, Tabuni A, Liu X (1997). Chloroquine induces human mononuclear phagocytes to inhibit and kill Cryptococcus neoformans by a mechanism independent of iron deprivation. J Clin Invest.

[27] Johnston SA, May RC (2010). The human fungal pathogen Cryptococcus neoformans escapes macrophages by a phagosome emptying mechanism that is inhibited by Arp2/3 complex-mediated actin polymerisation. PLoS Pathog.

[28] Geissmann F, Jung S, Littman DR (2003). Blood monocytes consist of two principal subsets with distinct migratory properties. Immunity..

[29] Cools-Lartigue Jonathan, Spicer Jonathan, McDonald Braedon, Gowing Stephen, Chow Simon, Giannias Betty, Bourdeau France, Kubes Paul, Ferri Lorenzo (2013). Neutrophil extracellular traps sequester circulating tumor cells and promote metastasis. Journal of Clinical Investigation.

[30] van Spil WE, Nooijen S, de Jong PY, Aliredjo RP, de Sevaux RG, Verhave JC (2015). Cryptococcal meningitis. Ned Tijdschr Geneeskd.

[31] Shi M, Mody CH (2016). Fungal infection in the brain: what we learned from Intravital imaging. Front Immunol.

[32] Kaufman-Francis K, Djordjevic JT, Juillard PG, Lev S, Desmarini D, Grau GER (2018). The early innate immune response to, and phagocyte-dependent entry of, Cryptococcus neoformans map to the perivascular space of cortical post-capillary Venules in Neurocryptococcosis. Am J Pathol.

[33] Alvarez M, Burn T, Luo Y, Pirofski LA, Casadevall A (2009). The outcome of Cryptococcus neoformans intracellular pathogenesis in human monocytes. BMC Microbiol.

[34] Pillay J, den Braber I, Vrisekoop N, Kwast LM, de Boer RJ, Borghans JAM (2010). In vivo labeling with (H2O)-H-2 reveals a human neutrophil lifespan of 5.4 days. Blood..

[35] Zhang DC, Chen G, Manwani D, Mortha A, Xu CL, Faith JJ (2015). Neutrophil ageing is regulated by the microbiome. Nature..

[36] Nordenfelt P, Tapper H (2011). Phagosome dynamics during phagocytosis by neutrophils. J Leukoc Biol.

[37] Fu MS, Coelho C, De Leon-Rodriguez CM, Rossi DCP, Camacho E, Jung EH (2018). Cryptococcus neoformans urease affects the outcome of intracellular pathogenesis by modulating phagolysosomal pH. PLoS Pathog.

[38] Chrisman CJ, Alvarez M, Casadevall A (2010). Phagocytosis of Cryptococcus neoformans by, and nonlytic exocytosis from, Acanthamoeba castellanii. Appl Environ Microbiol.

[39] Watkins RA, Andrews A, Wynn C, Barisch C, King JS, Johnston SA (2018). Cryptococcus neoformans escape from Dictyostelium Amoeba by both WASH-mediated constitutive exocytosis and Vomocytosis. Front Cell Infect Microbiol.

[40] Bouma G, Ancliff PJ, Thrasher AJ, Burns SO (2010). Recent advances in the understanding of genetic defects of neutrophil number and function. Brit J Haematol.

