# Supplementary material for: Nonlytic exocytosis of Cryptococcus neoformans from neutrophils in the brain vasculature
Source: Cell Commun Signal. 2019 Sep 9;17:117. doi: 10.1186/s12964-019-0429-0 (PMC6734394; doi:10.1186/s12964-019-0429-0)
Supplement: Supplementary file 1 — Table S1. Antibodies in the study. (DOCX 14 kb) [file 12964_2019_429_MOESM1_ESM.docx]

Additional file 1: Table S1 Antibodies in the study

| Antigen | Conjugate | Clone | Supplier | Catalog number | Cell type |
| --- | --- | --- | --- | --- | --- |
| Mouse F4/80 | PE | BM8 | eBiosicience | 12-4801-82 | Macrophage |
| Mouse Gr-1 (Ly6G/Ly6C) | Alexa Fluor 647 | RB6-8C5 | Invitrogen | RM3021 | Macrophage and neutrophil |
| Cryptococcal polysaccharide | None | E1 | Dr. F. Dromer | Not available | Cryptococcus neoformans |
| Mouse collagen IV | None | Polyclonal antibody | Invitrogen | PA1-26148 | Extracellular martix |
| Mouse Ly6G | None | 1A8 | Biolegend | 127602 | Neutrophil |
| Rabbit IgG | Alexa Fluor 647 | Polyclonal antibody | Invitrogen | A-21244 | Secondary antibody |
| Mouse IgG | Alexa Fluor 488 | Polyclonal antibody | Invitrogen | A-11001 | Secondary antibody |
| Rat IgG | Alexa Flour 555 | Polyclonal antibody | Invitrogen | A-21434 | Secondary antibody |
