# Supplementary figures and images for: Nonlytic exocytosis of Cryptococcus neoformans from neutrophils in the brain vasculature
Source: Cell Commun Signal. 2019 Sep 9;17:117. doi: 10.1186/s12964-019-0429-0 (PMC6734394; doi:10.1186/s12964-019-0429-0)

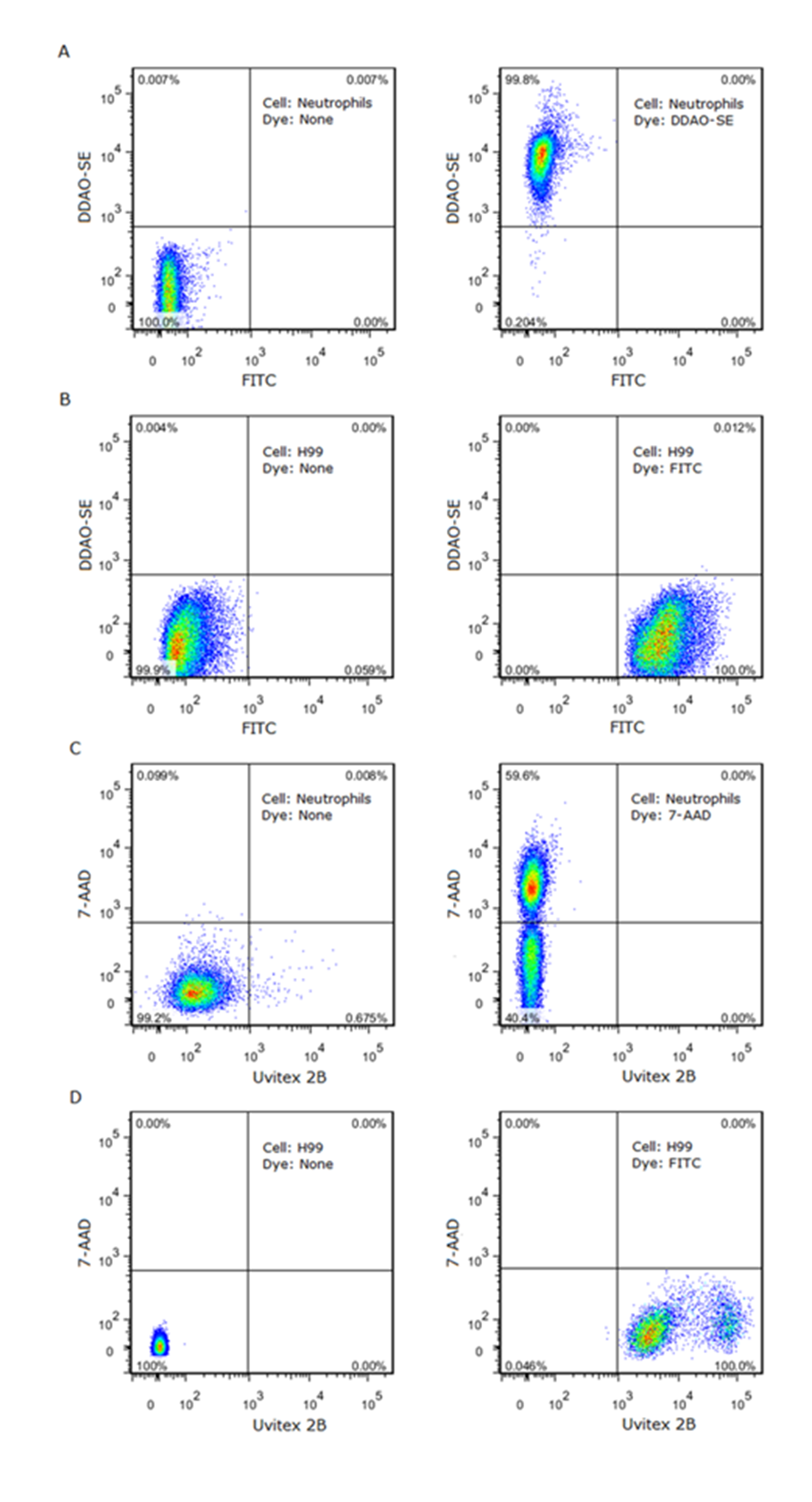

Supplement: Supplementary file 2 — Figure S1. Flow cytometry analysis of neutrophils and C. neoformans stained with various fluorophores. Bone marrow-derived neutrophils were stained with DDAO-SE (0.5 μM, Invitrogen) in PBS for 10 min and washed 3 times. C. neoformans cells were stained with FITC (1 mg/ml, Sigma-Aldrich) in PBS for 15 min and washed 3 times. Furthermore, neutrophils were stained with 7-AAD (1 μg/ml, Invitrogen) for 5 min to detect cell death, and C. neoformans were stained with Uvitex 2B (0.01%, Polysciences Inc.) in PBS for 1 min to detect extracellular fungi. Representative results of 5 repeated experiments. (TIF 575 kb) [file 12964_2019_429_MOESM2_ESM.tif]

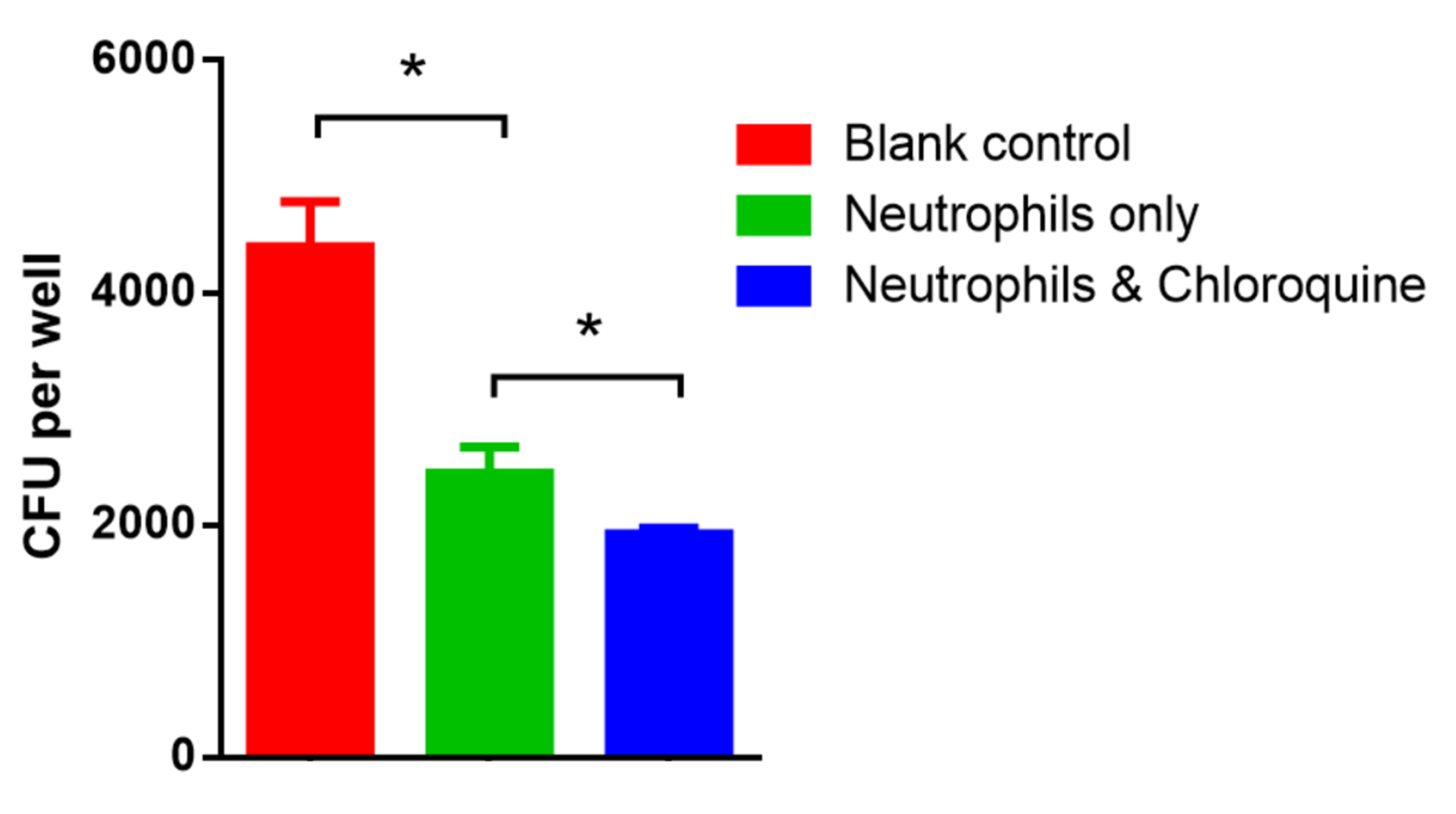

Supplement: Supplementary file 3 — Figure S2. Chloroquine increased the fungicidal activity of neutrophils against C. neoformans. In a 96-well plate, C. neoformans (5 × 10^3^ cells per well) were incubated alone or with neutrophils (1 × 10^5^ cells per well) and chloroquine (10 μM, working concentration) for 4 h. After incubation, the live fungi were quantified on YPA agar. *, p < 0.05. (TIF 235 kb) [file 12964_2019_429_MOESM3_ESM.tif]
